# A Treatise on Diseases of Infancy and Childhood

**Published:** 1886-12

**Authors:** 


A TREATISE ON DISEASES OF INFANCY AND CHILDHOOD. By J. Lewis Smith, M. D., Clinical Professor of Diseases of Children in Bellevue Hospital Medical College; Physician to Charity Hospital; Physician to the N. Y. Foundling Asylum; Consulting Physician to the N. Y. Infant Asylum; Consulting Physician to the class of Children's Diseases Bureau for the relief of the out-door poor, Bellevue. Sixth edition, thoroughly revised, with forty illustrations, pp. 870. Lea Brothers & Co., Philadelphia. 1886.

It is a pleasure to the busy practitioner—interested in the advancement of the science of his profession—to meet, fresh from the hands of its author, a medical classic such as the one presented in Smith on Diseases of Children.

Those familiar with the former editions of the work will readily recognize the painstaking with which this revision has been made. Many of the articles treating of some of the more formidable maladies incident to infancy and childhood have been entirely re-written. In this edition, in its etiology, pathology and treatment, it is brought full abreast with the present advancement of pathology and therapeutics; yet, as applied to many maladies, he gives as boldly and candidly his own peculiar views, based upon an extended experience reaching back through many years of active practice, both private and public. In fact, the whole work is enriched with a research and reasoning which plainly show that the author has spared neither time nor labor in bringing it to its present approach towards perfection. The extended table of contents and the well prepared index will enable the busy practitioner to reach readily and quickly for reference the various subjects treated of in the body of the work, and even those who are familiar with former editions will find the improvements in the present richly worth the cost of the work.

The mechanical execution of the work is in the usual good style of the well-known house of the Leas, familiar to all American medical readers.

